# On the Precise Seat of the Variolous Pustule

**Published:** 1829-09

**Authors:** George Oakley Heming

**Affiliations:** Surgeon; Member of the Royal College of Surgeons.


### THE LONDON
### Medical and Physical Journal.

No. 367, VOL. LXII.] SEPTEMBER, 1829. [No. 39, *New Series*.

For many fortunate discoveries in medicine, and for the detection of numerous errors, the world is indebted to the rapid circulation of Monthly Journals; and there never existed any work, to which the Faculty, in Europe and America, were under deeper obligations than to the *Medical and Physical Journal of London*, now forming a long, but an invaluable series.—RUSH.

### ORIGINAL PAPERS, AND CASES,
### OBTAINED FROM PUBLIC INSTITUTIONS AND OTHER AUTHENTIC SOURCES.

### VARIOLA.

*On the precise Seat of the Variolous Pustule.* By Mr. George Oakley Heming, Surgeon; Member of the Royal College of Surgeons.

It has appeared to me that a distinction may be made between the pustule of variola and the vesicle of varicella, by observing the distinct seat of these affections. From an investigation pursued with considerable care, I think I have ascertained that the former has its seat in the sebaceous glands and mucous follicles, whilst the latter seems to be merely subcuticular in general.

The exact seat of the variolous pustule seems to be determined by observing the textures most apt to be affected by it, and the textures excluded from it; the form of the pustule itself, its difference from that of the vesicle of varicella, and its similarity to that of some other affections of the sebaceous glands.

A still more direct proof of this point flows from minutely observing the anatomy of the sebaceous glands and mucous follicles, and tracing that of the variolous pustule.

The practical advantages of the inquiry consist in its affording a source of diagnosis, and in its tendency, in this manner, to settle some disputes which still divide the medical profession, and to enable us better to decide upon the real value of vaccination.

It may be observed that the variolous pustule is confined to the skin and mucous membranes. After much diligent search, I have never been able to detect any thing at all resembling it in the serous membranes. I do not mean to say that there is, in no case of variola, inflammation of a serous membrane, but that I have not been able to detect any appearance of variolous pustule, or difference from that of common inflammation. Then the circular, flat, and indented form of the variolous pustule differs widely from the hemispherical form of the vesicle of varicella: it is obvious, too, from the hardness felt on an early examination, that deeper seated parts are affected in the former than in the latter disease.

Before I proceed, I would observe that, although I have spoken of the variolous pustule as affecting the follicles of the mucous membrane, such pustules are never perfect; the presence of a cuticle being required to form the perfect pustule. The variolous affection of the mucous membrane assumes, first, the form of an inflamed point, then becomes an ulcer, and then passes into a state resembling that in aphthæ. Wrisberg, Contumnius, and others, may therefore well have disputed whether the perfect pustule of variola existed internally.

It is well known that the variolous pustule occurs in every part of the surface of the body. Haller considered that the sebaceous glands had not been demonstrated in every part. Morgagni had seen them in the back, neck, and other parts. Bichat goes so far as to doubt the existence of the sebaceous glands; but his follower in this inquiry, Beclard, distinctly affirms their existence. Lastly, Chevalier says that they exist in every part of the cutaneous texture. The last-named author has deposited preparations in the museum of the College of Surgeons, showing the sebaceous glands in the nose and chin of the infant. He contends, in his Lectures delivered before the College, which have been since published, that there are two sets of these glands, one more superficial than the other.

The variolous affection is to be seen in some part of the track of the mucous membrane, in almost every case of the disease; but in no single case in great number. It is equally true that the mucous follicles pervade the whole of these membranes. There are some parts of the mucous membranes, as on the tongue, the palate, and the mouth generally, covered by a cuticle of sufficient thickness occasionally to allow of being distended by fluid effused underneath, and, consequently, of the formation of a pustule. But, in most parts of the mucous membranes, there is either no cuticle, or it is so thin as not to allow of distention by the subjacent effusion of fluid: in these, of course, no pustule can be formed; but we observe the mucous follicle enlarged by inflammation, covered by a layer of whitish matter, very much resembling that in aphthæ, and sometimes ulcerated. Whether one or other of these appearances be found, will depend upon the different periods of the disease at which the examination of the mucous membranes takes place. It is a curious fact that, throughout the mucous lining of the bowels, extending from the stomach to the rectum, there is no portion of it where the mucous follicles are so frequently affected by smallpox as in that of the appendix vermiformis.

In regard to any affection of a serous membrane, I must repeat that I have never observed any thing either pustular or of the character of the affection of the follicles of the mucous membrane, which I have just described.

The sebaceous glands, as is well known, are small bodies whose office is to secrete a greasy matter, which is poured forth by their excretory ducts, and distributed over the skin; and into each of these ducts the cuticle dips. This organization cannot be discerned in the healthy state of the sebaceous glands; but, when they are diseased, it may sometimes be seen even without a lens. They are very prone to diseases, of which one form is called *acne.* It was the resemblance that this diseased state of the sebaceous glands bears to the little tumors found in the early stage of smallpox, and the striking similitude to a smallpox pustule at a more advanced period, when an herpetic eruption about the chin extends over an enlarged sebaceous gland, conjoined to other circumstances, which first led me to suppose that the sebaceous glands and mucous follicles were the parts affected by variola.

Sir A. Cooper remarks, that some tumors arise from an enlargement of the sebaceous cysts, in consequence of their orifices being obstructed; and he observes, that "within the cyst there is a lining of cuticle, which adheres to its interior, and several desquamations of the same substance are found within the first lining." I am now attending a young woman who has a disease of these glands, and the orifices are so much enlarged that I can pass into them a bristle. I applied a blister, and, by this means removing the cuticle, had a drawing taken of the part, in which this fact is illustrated. The sebaceous glands and mucous follicles bear the strictest analogy to each other, both in their structure and functions, and consequently are apt to be affected by the same diseases.

I now proceed to give an account of the appearances of the perfect variolous pustule: I would first observe, most particularly, that, although the indentation of the pustule of smallpox has generally been considered by medical writers as one among many other circumstances by which we may be enabled to distinguish it from chickenpox, it appears to me that, not being acquainted with the cause of this very curious circumstance, they have not attached to it the importance which it seems to demand. This indentation in the pustule can only depend upon the structure of the part affected; it is the natural formation of the cuticle at that part which produces the depression in its centre.

Dr. Armstrong says, "I have never seen the central depression absent in smallpox, and, what is remarkable, I have never seen it present in chickenpox." My own practice confirms this observation; and I think that most medical men must have observed the uniformity of the central depression in smallpox. The inference I would draw is, that smallpox at all times attacks the same structure.

At the earliest stages of the eruption of smallpox, it is generally first seen in the hands and face, where small red spots indicate the inflamed state of the cutis. On these spots a small, round, hard tumor may be perceived by the touch, before it becomes visible. In twenty-four hours, it is still more distinct. It gradually changes its form until the third or fourth day, when it is perfectly circular, with a flattened top, in the centre of which an indentation may be perceived, resembling, it has been remarked, "the impression made in the skin with the head of a large pin." The vesicle is then about the eighth part of an inch in diameter; it is of a cellular structure, and filled with lymph somewhat turbid, and finally purulent. By the fifth or sixth day, its size has augmented to twice its former diameter. The central depression is commonly evident on the second or third day in some of the pocks, where they are numerous. Dr. Munro, in his Observations on the Smallpox, remarks, that "the central clear part of the pimple is evidently depressed on the fourth or fifth day: this depression is not to be perceived in all the pimples in the same light; but, by turning the body, it can be seen in those vesicular pimples in which it had not been previously perceptible. This fact is generally overlooked, and has often led to the denial of the existence of the central depression when it was present."

There may be cases in which the central depression is not perceived without much difficulty; but, if the pustule be carefully examined by a microscope, and in a proper light, it will be discovered. It is most manifest when the internal fluid is clear, and is essentially different from the depression in other eruptions, which exist only after the apex is encrusted. As the disease advances, a red ring shows itself round the circumference of the pustule, and becomes wider as it increases in size. There is a remarkable appearance of the pustule on the sixth or seventh day, which was pointed out to me by Dr. Marshall Hall: there is an external ring of rose colour, in which is another ring of white, evidently rendered so by the colour of the contained fluid; within this is a third ring, which is red, and has an appearance as if the surface of the pustule was in contact with the flesh beneath; and in the middle of this there is a portion which again looks white, but is dull and cloudy. These appearances I have constantly observed about the sixth or seventh day. After the seventh or eighth day, the pustule loses its indented character, and becomes nearly spherical. If it be opened, it will be found to contain pus; and not only the small sebaceous gland, which was at first merely inflamed and enlarged, has become disorganized, but all these small glands within the circumference of the pustule have partaken of this disorganization, and a slough is formed nearly of the size of the base of the pustule. A portion of coagulable lymph is thrown out around the slough; and this I am inclined to think is what Mr. Cruikshank supposed to be a membrane situated between the rete mucosum and cutis, and which he has called the membrane of smallpox.

Mr. Cruikshank describes this vascular membrane as situated between the rete mucosum and cutis, and which he had injected in the skin of persons who had died of the smallpox. During the summer months he macerated in water pieces of smallpox skin, which had been kept for some time in spirits, and he says "the cuticle and rete mucosum were turned down, and, upon the eighth or ninth day, I found I could separate a vascular membrane from the cutis." There is little doubt but this was the vascular network described by Bichat, which Mr. Cruikshank had injected, and, in consequence of the effusion of lymph which I have previously described, he was enabled to separate it in the form of a membrane.

From the back of a patient who died of the smallpox I removed a portion of skin covered with pustules, which I macerated in water eight or ten days. I succeeded in removing the cuticle from the pustules; these still retaining their form, and being covered by another membrane. But, in the present doubtful state of our knowledge as to the existence of the rete mucosum in the white races, I found some difficulty in deciding whether this was the rete mucosum, or only a layer of coagulable lymph effused at an early period of the formation of the pustule, and subsequently raised with the cuticle by the pus contained in the pustule. Dr. Armstrong has this preparation.

Mr. Cruikshank found that in the centre of the pustule of smallpox there was a white substance, which he could not inject; and this Mr. Hunter said was a slough formed by the variolous inflammation. He thought it was always to be found in this disease, and that it was a circumstance by which it might be distinguished. In most cases it does exist, but I believe there are some exceptions. Upon this subject, however, I cannot speak decidedly, as I have never had an opportunity of minutely examining that kind of pustule. The cases to which I allude are those of modified smallpox, particularly as occurring after vaccination. Here we have an inflammation of a more moderate kind, and partaking more of the adhesive character. Lymph is poured out, which gives a peculiar hardness to the pustule, and, as the eruption subsides, a small tubercle is left. The lymph, however, is again absorbed, and the hardness and swelling are gradually removed. If these pustules were examined at any period, I do not think the slough would be found.

The parts around the nipple, particularly in the female, seem to afford the best place for the examination of the structure of the smallpox pustule, as the sebaceous glands there are more conspicuous than in most other parts of the body. In order to investigate it to the greatest advantage, it should be done at an early period of the eruption, and before the disorganization of the parts takes place.

If I have succeeded in showing that variola and varicella always attack different structures, I shall have established a fact which will be useful in any further investigation of this subject. If the seat of the smallpox be ascertained to be the sebaceous glands and mucous follicles, something not immaterial is added to our knowledge of the disease: there is a foundation laid for future inquiry.

There are many other points of difference between the variolous and varicellous affections, which are known to those who have considered this subject, and must not be overlooked; but I have been rather desirous to draw the attention to those differences which prove that the two diseases attack different structures.

The minute anatomy of the parts affected has been so neglected, that our knowledge of the progress of the variolous pustule is but imperfect; and this is a result of the importance of that knowledge not being thoroughly understood. The varicellous vesicle is hemispherical and inelastic; it is easily broken, and, being once opened, it empties itself entirely, and never fills again. The variolous pustule is circular and elastic, and, if an opening be made in it, and some matter be taken from it, the pustule will nevertheless again soon be distended as fully as before; and this is evidently a consequence of its cellular structure.

Since writing the above, I find that I have been anticipated in these views by M. Velpeau.* I cannot say that I do not regret this circumstance; but, in the midst of this regret, I have great satisfaction in finding the views themselves established on the firm basis of that gentleman's well known talent for pathological observation; and, as my mode of proceeding in this investigation has been rather different, I do not think I ought to suppress my own paper on this interesting subject. It is something to have thought and observed the same things with M. Velpeau.

*Kentish Town; August* 1829.

---

### HYSTERITIS PUERPERALIS.

* Bull. de Soc. Philomath. Juin 1825, and Bateman's Synopsis of Cutaneous Diseases, by Dr. A. T. Thomson, p. 269.

